# Association between the triglyceride-glucose index and risk of type 2 diabetes mellitus and the mediating effect of BMI: a comparative analysis in Chinese and Japanese populations

**DOI:** 10.3389/fendo.2026.1701371

**Published:** 2026-03-02

**Authors:** Yuxian Chen, Haiyong Zeng, Ziqi Luo, Haofei Hu, XinYu Wang

**Affiliations:** 1 Department of Endocrinology and Metabolism, The First Affiliated Hospital of Shenzhen University, Shenzhen, Guangdong, China; 2 School of Medicine Shenzhen University, Shenzhen, Guangdong, China; 3 Department of General Practice, Shenzhen Second People’s Hospital, Shenzhen, Guangdong, China; 4 Department of Nephrology, Shenzhen Second People’s Hospital, Shenzhen, Guangdong, China

**Keywords:** body mass index, east asian populations, mediation analysis, the triglyceride-glucose index, type 2 diabetes mellitus

## Abstract

**Objective:**

The triglyceride–glucose (TyG) index is a surrogate marker of insulin resistance and metabolic dysregulation. We aimed to examine its association with incident type 2 diabetes mellitus (T2DM) in Chinese and Japanese adults and to quantify the mediating role of body mass index (BMI). However, ethnic differences in the TyG—diabetes association, population-specific thresholds, and potential mediating mechanisms—remain unclear. This study aimed to evaluate the association between the TyG and incident T2DM in Chinese and Japanese adults and to quantify the mediating role of BMI in these associations.

**Methods:**

We conducted a retrospective cohort study using data from the China Rich Healthcare Group (n=199,050) and the Japanese NAGALA database (n=15,464). TyG was calculated as ln[fasting triglycerides (mg/dL) ×fasting plasma glucose(mg/dL)/2]. Incident T2DM was defined according to American Diabetes Association criteria. Multivariable Cox models, restricted cubic splines, and two-piecewise regression were used to characterize linear and nonlinear associations between TyG and diabetes risk. Predictive performance was assessed using receiver operating characteristic (ROC) curves. Mediation analysis with 5,000 bootstrap replications quantified the proportion of the TyG–diabetes association mediated by BMI.

**Results:**

During a median follow-up of 5.0 years, 2,563 participants developed T2DM. Recent reviews and experimental studies indicate that Japanese adults often develop type 2 diabetes at relatively modest levels of adiposity, frequently with insufficient insulin secretion and a disproportionately high burden of visceral and ectopic fat (fully adjusted HR per 1-unit increase, 2.32; 95% CI, 2.16–2.47), with clear gradients across TyG quartiles in both cohorts. BMI partially mediated the TyG–diabetes association (19.06% in Chinese vs 14.22% in Japanese adults), supporting adiposity-related pathways and population differences in metabolic mediation. Nonlinear analyses suggested cohort-specific inflection points, with risk rising more steeply above TyG ≈8.98 in Chinese and ≈7.88 in Japanese adults. TyG showed moderate discrimination for 5-year diabetes risk (AUC ≈0.74), outperforming triglycerides alone; fasting plasma glucose (FPG) remained more discriminative, and ROC results are reported descriptively.

**Conclusions:**

Higher baseline TyG was associated with incident T2DM in both Chinese and Japanese adults, with BMI partially mediating the TyG–diabetes association. These findings suggest that TyG captures triglyceride–glucose dysregulation beyond overall adiposity, with population-specific differences in metabolic pathways. The identification of nonlinear patterns underscores the need for population-tailored risk stratification based on TyG.

## Introduction

Type 2 diabetes mellitus (T2DM) is a rapidly expanding global public health concern, with more than 589 million adults affected in 2024 and projections exceeding 850 million by 2050. East Asia bears a disproportionate share of this burden: China currently has the highest national prevalence worldwide, while Japan continues to experience a growing incidence despite comparatively lower adiposity levels (1). These trends highlight the complex metabolic vulnerabilities within East Asian populations, where diabetes often develops at younger ages and at lower body mass index (BMI) thresholds compared to Western populations (2).

Insulin resistance (IR) is a central mechanism in the pathogenesis of T2DM (3). However, measuring IR directly using gold-standard methods is impractical for routine clinical or large-scale epidemiologic use. The triglyceride-glucose (TyG) index—calculated from fasting triglyceride (TG) and fasting plasma glucose (FPG) levels—has emerged as a simple and reliable surrogate marker of IR (4, 5). Previous studies have shown that higher TyG values are associated with increased risks of metabolic syndrome, cardiovascular disease, and metabolic dysfunction–associated steatotic liver disease (MASLD) (6–9), supporting its role as an early indicator of metabolic dysregulation.

Several cohort studies support the predictive value of the TyG for diabetes. In a 5-year prospective study of prediabetic adults, individuals in the highest TyG quartile (Q4) exhibited a substantially higher risk of progressing to T2DM (10). Moreover, research in Japanese adults confirmed a significant association between higher baseline TyG and incident T2DM, showing a U-shaped pattern (11). Together, these findings highlight the potential of the TyG as an early metabolic risk indicator across diverse East Asian populations.

Beyond reflecting glucose–lipid dysregulation, accumulating evidence indicates that TyG is associated with adiposity-related metabolic disturbances (12). Individuals with elevated TyG values consistently exhibit greater visceral adiposity, and TyG has emerged as an independent predictor of visceral obesity across diverse populations (13, 14). These observations indicate that the TyG captures adiposity-related metabolic dysfunction that contributes to insulin resistance, underscoring the relevance of incorporating adiposity indicators—such as BMI—when elucidating the TyG–diabetes relationship.

Recent Mendelian randomization evidence further shows that East Asian adults may develop insulin resistance even at comparatively low BMI levels due to their limited β-cell secretory capacity and heightened metabolic susceptibility (15). This dissociation between BMI and metabolic dysfunction highlights that excess metabolic risk can arise independently of overall adiposity. As a result, BMI represents only a partial proxy for total and central adiposity and cannot fully capture the adiposity-related metabolic burden reflected by TyG. Although more precise measures of abdominal adiposity—such as waist circumference, waist–hip ratio, or imaging-derived visceral fat—would offer deeper mechanistic resolution, these variables were not collected consistently or comparably across the two cohorts. Given these constraints, BMI was the only adiposity metric measured in a harmonized manner in both datasets and therefore served as the most appropriate and feasible mediator for evaluating the TyG–diabetes relationship in this study.

Despite the growing evidence linking TyG to diabetes risk, important gaps remain. Most existing studies were conducted within single cohorts, applied heterogeneous analytic approaches, and did not evaluate population differences, limiting their comparability and generalizability. Moreover, although the TyG clearly reflects adiposity-related metabolic dysfunction, the extent to which BMI mediates the TyG–diabetes association has not been quantified, particularly in East Asian populations where metabolic risk may arise at lower BMI thresholds. Whether the TyG–diabetes relationship exhibits population-specific patterns or nonlinear associations also remains insufficiently understood.

Therefore, we conducted an analysis of two large retrospective cohorts to (1) evaluate the association between the TyG and incident T2DM in Chinese and Japanese adults; (2) characterize population-specific association patterns, including potential nonlinearities; and (3) quantify the mediating role of BMI in the TyG–diabetes relationship within each population. By addressing these gaps, this study provides a more rigorous and comparable evaluation of the TyG and clarifies how adiposity pathways contribute to diabetes risk across East Asian populations.

## Methods

### Study design and data sources

This retrospective cohort study evaluated the association between the triglyceride–glucose (TyG) index and incident type 2 diabetes mellitus (T2DM) in two East Asian populations. Data were derived from the China Rich Healthcare Group cohort and the Japanese NAGALA (NAFLD in the Gifu Area, Longitudinal Analysis) cohort, both obtained as fully de-identified datasets from the public DataDryad repository (16, 17).

Ethical approval for the Chinese cohort was obtained from the Rich Healthcare Group Ethics Committee, while the Japanese cohort was approved by the Murakami Memorial Hospital Ethics Committee. In the original data descriptions, the investigators explicitly authorized public sharing of de-identified data for non-commercial research and scientific publication via the DataDryad repository. Both cohorts complied with the Declaration of Helsinki (18). As the present analysis used de-identified secondary data, no additional institutional review was required.

The Chinese dataset consisted of health examination records collected from 32 sites across 11 major cities between 2010 and 2016. The Japanese dataset included adults undergoing routine health check-ups at Murakami Memorial Hospital between 2004 and 2015. Within each cohort, anthropometric and laboratory measurements followed standardized clinical protocols.

### Study population

**Figure 1:**
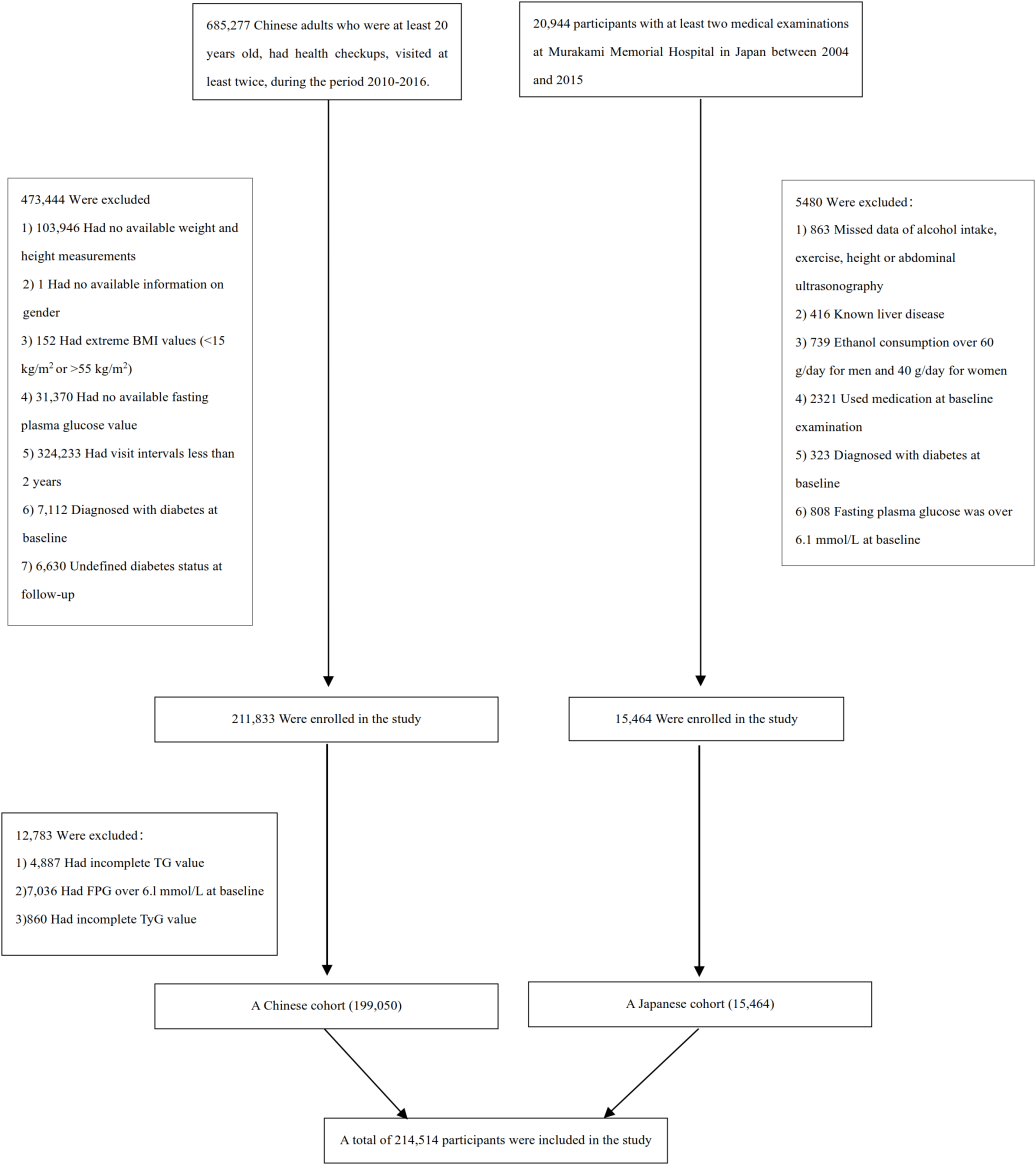
Participant flowchart. Flow diagram showing participant selection in the Chinese and Japanese cohorts. Exclusions included missing TyG components, missing follow-up data, baseline T2DM, extreme BMI values, excessive alcohol intake (Japanese cohort), viral hepatitis, medication use, or follow-up <2 years. A total of 214,514 participants (199,050 Chinese; 15,464 Japanese) were included in the final analyses.

A total of 685,277 Chinese and 20,944 Japanese adults were initially screened. Participants were excluded for missing baseline triglyceride or fasting glucose values, missing follow-up, baseline diabetes, or follow-up <2 years. Additional exclusions in the Japanese cohort included excessive alcohol intake, viral hepatitis, medication use, and incomplete questionnaire data. Implausible BMI values (<15 or >55 kg/m²) were excluded. After applying cohort-specific criteria, 199,050 Chinese and 15,464 Japanese adults were included (**Figure 1**). This study is related to our previously published study by the same authors on AIP and incident T2DM (19). The current submission focuses on TyG as a distinct exposure and evaluates its population-specific non-linear association patterns and BMI mediation. Although both analyses used the China Rich Healthcare Group and Japanese NAGALA cohorts, the final analytic populations are not identical due to exposure-specific data requirements and exclusion criteria.

### Exposure: the triglyceride-glucose index

Baseline TyG was calculated using the established formula: ln[fasting triglycerides (mg/dL) ×fasting plasma glucose(mg/dL)/2]. Values measured in mmol/L were converted to mg/dL using standard conversion factors. TyG was analyzed as a continuous variable and by ethnicity-specific quartiles.

### Outcome: incident diabetes during follow-up

Our primary outcome variable was incident diabetes during follow-up (dichotomous variable: 0 = no incident diabetes, 1 = incident diabetes). Incident DM was defined according to the American Diabetes Association (ADA) criteria (20). Participants were considered to have developed diabetes during follow-up if any of the following criteria were met: fasting plasma glucose (FPG) ≥7.0 mmol/L, hemoglobin A1c (HbA1c) ≥6.5%, a documented physician diagnosis of diabetes within the dataset, or self-reported diabetes confirmed by available clinical or laboratory information.

### Covariates

Baseline covariates included age; sex; body mass index (BMI); systolic and diastolic blood pressure (SBP, DBP); smoking status; alcohol intake; fasting plasma glucose (FPG); total cholesterol (TC); triglycerides (TG); low-density lipoprotein cholesterol (LDL-c); high-density lipoprotein cholesterol (HDL-c); alanine aminotransferase (ALT); and aspartate aminotransferase (AST). Covariates were selected based on their established or plausible associations with diabetes risk and TyG values (21, 22).

Smoking status was classified as current smoker versus non-smoker. Alcohol intake was categorized as drinker versus non-drinker based on questionnaire data. BMI was calculated as weight (kg) divided by height squared (m²) and categorized according to Chinese criteria (<18.5, 18.5–24.0, 24.0–28.0, and ≥28.0 kg/m²).

### Missing data

Missing data were addressed using multiple imputation by chained equations (MICE) (23), assuming missing at random (MAR) (24). Five imputed datasets were generated, including all variables used in the main analyses (age, sex, BMI, blood pressure, lipid profile, liver enzymes, fasting glucose, smoking, and alcohol intake). Estimates from Cox models were pooled across imputations using Rubin’s rules. Results from complete-case analyses were similar and are not shown.

### Statistical analysis

Baseline characteristics across TyG quartiles were compared using ANOVA or the Kruskal-Wallis test for continuous variables and the χ² test for categorical variables. Multicollinearity was evaluated using the variance inflation factor (VIF) (25). Variables with VIF >5 were considered to have significant collinearity and were excluded from multivariable models (**Supplementary Table 1**). Because TC showed substantial collinearity in the initial model (VIF = 8.6), TC was removed and VIFs were recalculated. Cox proportional hazards models were used to estimate hazard ratios (HRs) and 95% confidence intervals (CIs) for the association between TyG and incident T2DM. Three models were fitted: Model I was unadjusted; Model II was adjusted for age, sex, BMI, smoking status, drinking status, SBP, and DBP; and Model III was additionally adjusted for HDL-c, LDL-c, ALT, and AST. Because FPG and TG are components of the TyG, they were not additionally adjusted for in multivariable models to avoid over-adjustment and collinearity. TyG was analyzed as a continuous variable and by ethnicity-specific quartiles. Proportional hazards assumptions were assessed using Schoenfeld residuals. Restricted cubic spline models were fitted to evaluate potential nonlinear associations between TyG and T2DM risk. When nonlinearity was detected, a two-piecewise Cox model was fitted, and model fit was compared using likelihood ratio tests. Prespecified subgroup analyses were conducted according to age, sex, BMI category, blood pressure category, smoking status, drinking status, and ethnicity.

Continuous variables were categorized using clinical cutoffs: age (<45, 45–60, ≥60 years), BMI (<18.5, 18.5–24, 24–28, ≥28 kg/m²), SBP (<140/≥140 mmHg), and DBP (<90/≥90 mmHg) (26). Interaction terms were assessed using likelihood ratio tests. Sensitivity analyses excluded participants who smoked, consumed alcohol, had obesity (BMI ≥28 kg/m²), or had hypertriglyceridemia (TG ≥1.7 mmol/L). TyG quartiles were also examined to evaluate consistency with continuous analyses. Predictive performance was assessed using receiver operating characteristic (ROC) curves for 5-year T2DM risk; AUC values were compared using the DeLong method, and optimal cutoffs were determined using the Youden index. Mediation analysis quantified the proportion of the TyG–T2DM association mediated by BMI using natural direct and indirect effects with 5,000 bootstrap replications based on the counterfactual framework (27). Analyses were performed overall and stratified by ethnicity. All analyses were conducted in R 4.2 and EmpowerStats, with two-sided P <0.05 considered statistically significant.

## Results

### Baseline characteristics of participants

The baseline characteristics of the study population are presented in **Table 1**. A total of 214,514 adults were included in the final analysis, comprising 199,050 Chinese and 15,464 Japanese participants. The mean baseline TyG was 8.33 ± 0.61, the mean age was 41.95 ± 12.27 years, and 54.29% of participants were male. During a median follow-up of 5.0 years, 2,563 individuals (1.19%) developed type 2 diabetes.

**Table 1:** Baseline characteristics of participants across TyG quartiles.

TyG group	Q1 (<7.9)	Q2 (7.9-8.3)	Q3 (8.3-8.7)	Q4 (≥8.7)	P-value
Participants	52852	53416	53615	54631	
Age (years)	37.8 ± 9.8	40.5 ± 11.7	43.4 ± 12.9	46.0 ± 12.8	<0.001
Gender, n (%)					<0.001
Male	16957 (32.1%)	25481 (47.7%)	32879 (61.3%)	41145 (75.3%)	
Female	35895 (67.9%)	27935 (52.3%)	20736 (38.7%)	13486 (24.7%)	
Country, n (%)					<0.001
Chinese participants	46235 (87.5%)	49811 (93.3%)	50636 (94.4%)	52368 (95.9%)	
Japanese participants	6617 (12.5%)	3605 (6.7%)	2979 (5.6%)	2263 (4.1%)	
Smoking status, n (%)					<0.001
Non-smoker	46545 (88.1%)	43844 (82.1%)	40056 (74.7%)	34779 (63.7%)	
Smoker	6307 (11.9%)	9572 (17.9%)	13559 (25.3%)	19852 (36.3%)	
Drinking status, n (%)					<0.001
Non-drinker	47649 (90.2%)	46301 (86.7%)	44461 (82.9%)	41937 (76.8%)	
Drinker	5203 (9.8%)	7115 (13.3%)	9154 (17.1%)	12694 (23.2%)	
BMI (kg/m²)	21.1 ± 2.6	22.3 ± 2.9	23.6 ± 3.1	25.2 ± 3.1	<0.001
SBP (mmHg)	111.9 ± 13.9	116.1 ± 15.1	120.2 ± 15.9	125.1 ± 16.4	<0.001
DBP (mmHg)	69.6 ± 9.5	72.2 ± 10.0	74.8 ± 10.5	78.3 ± 10.9	<0.001
FPG (mmol/L)	4.7 ± 0.5	4.8 ± 0.5	5.0 ± 0.5	5.1 ± 0.5	<0.001
TC (mmol/L)	4.3 ± 0.8	4.6 ± 0.8	4.8 ± 0.9	5.2 ± 0.9	<0.001
TG (mmol/L)	0.6 (0.5-0.6)	0.8 (0.8-0.9)	1.2 (1.1-1.4)	2.1 (1.7-2.7)	<0.001
HDL-c (mmol/L)	1.5 ± 0.3	1.4 ± 0.3	1.3 ± 0.3	1.2 ± 0.3	<0.001
LDL-c (mmol/L)	2.5 ± 0.6	2.7 ± 0.7	2.9 ± 0.7	3.0 ± 0.7	<0.001
ALT (U/L)	13.9 (10.9-18.4)	16.0 (12.0-22.5)	19.0 (14.0-28.0)	25.8 (18.0-38.7)	<0.001
AST (U/L)	19.9 (16.0-24.1)	20.9 (17.0-25.9)	22.2 (18.0-27.8)	25.0 (20.0-31.6)	<0.001
TyG	7.6 ± 0.2	8.1 ± 0.1	8.5 ± 0.1	9.1 ± 0.4	<0.001

Values are presented as n (%), mean ± SD, or median (interquartile range) as appropriate.

BMI, body mass index; FPG, fasting plasma glucose; DBP, diastolic blood pressure; TC, total cholesterol; SBP, systolic blood pressure; TG, triglyceride; ALT, alanine aminotransferase; LDL-c, low-density lipid cholesterol; AST, aspartate aminotransferase; HDL-c, high-density lipoprotein cholesterol; TyG, triglyceride–glucose index.

Across increasing TyG quartiles, participants tended to be older, more likely to be male, and more likely to report smoking or alcohol consumption. BMI, SBP, DBP, FPG, TC, TG, LDL-c, ALT, and AST increased across quartiles, whereas HDL-c decreased (all P < 0.001). These gradients were observed in both Chinese and Japanese. Additional differences in adiposity, lipid profiles, and lifestyle factors between the two populations are summarised in **Supplementary Table 2**.

### The incidence rate of diabetes

T2DM incidence increased across TyG quartiles in the overall cohort and within each ethnic group (**Table 2**). In the total population, incidence rose from 0.3% in Q1 to 2.7% in Q4 (P for trend < 0.001). Among Chinese, the incidence increased from 0.2% to 2.5% across Q1–Q4, and among Japanese from 1.2% to 7.4%. This pattern indicates a clear gradient in T2DM risk with higher TyG, with a steeper absolute increase in the Japanese cohort.

**Table 2:** Incidence rate of incident diabetes.

TyG	Participants(n)	Diabetes events(n)	Incidence rate (95% CI) (%)	Cumulative incidence (per 1,000 person-year)
All participants				
Total	214514	2563	1.1(1.1-1.2)	3.57
Q1 (<7.9)	52852	152	0.3(0.2-0.3)	0.81
Q2 (7.9-8.3)	53416	303	0.5(0.5-0.6)	1.65
Q3 (8.3-8.7)	53615	613	1.1(1.1-1.2)	3.35
Q4 (≥8.7)	54631	1495	2.7(2.6-2.9)	8.17
P for trend			<0.001	
Chinese participants				
Total	199050	2190	1.1(1.1-1.2)	3.51
Q1 (<7.9)	46235	103	0.2(0.1-0.3)	0.68
Q2 (7.9-8.3)	49811	244	0.4(0.4-0.5)	1.52
Q3 (8.3-8.7)	50636	515	1.0(0.9-1.1)	3.55
Q4 (≥8.7)	52368	1328	2.5(2.4-2.7)	8.02
P for trend			<0.001	
Japanese participants				
Total	15464	373	2.4(2.2-2.7)	3.98
Q1 (<7.9)	6617	49	0.7(0.5-0.9)	1.26
Q2 (7.9-8.3)	3605	59	1.6(1.2-2.1)	2.60
Q3 (8.3-8.7)	2979	98	3.2(2.3-3.9)	5.70
Q4 (≥8.7)	2263	167	7.4(6.3-8.5)	11.50
P for trend			<0.001	

### TyG distribution by incident diabetes during follow-up

**Figure 2:**
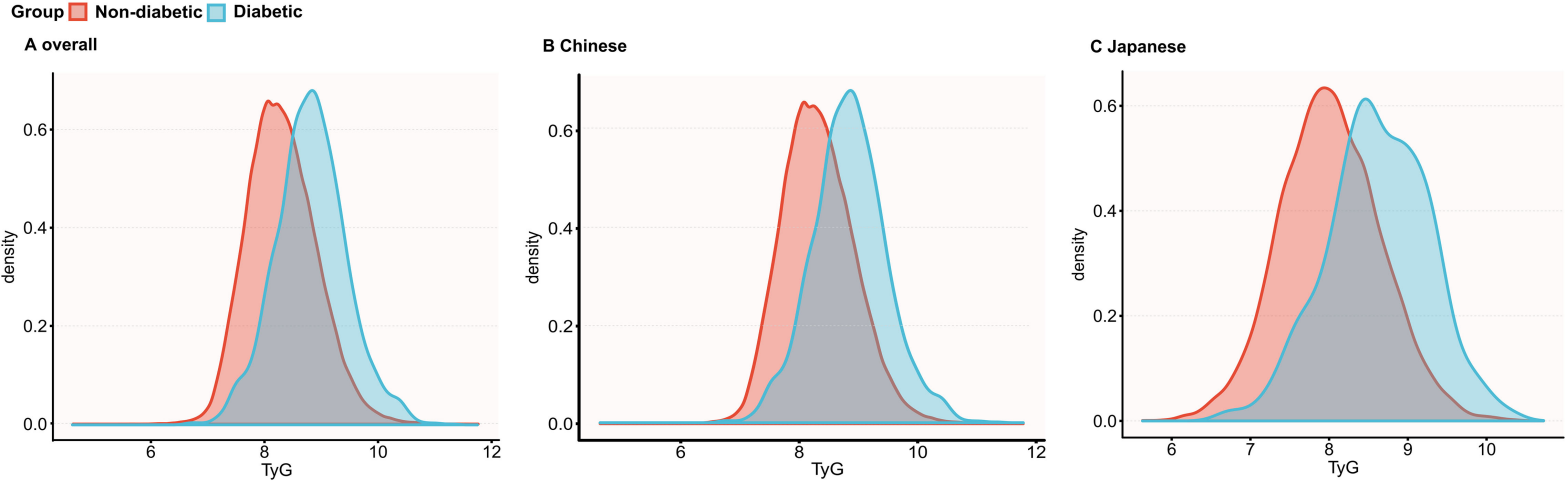
TyG density curves by diabetes status. Kernel density curves illustrating the distribution of TyG in non-diabetic and diabetic individuals in **(A)** the total population, **(B)** the Chinese cohort, and **(C)** the Japanese cohort.

**Figure 2** presents the baseline TyG density curves stratified by incident diabetes during follow-up. In both cohorts, the curves for those who developed diabetes shifted to the right, indicating higher baseline TyG levels compared to those who did not. The overlap was more pronounced in the Chinese cohort than in the Japanese cohort.

**Supplementary Table 3** shows baseline TyG values stratified by incident diabetes. In the Chinese cohort, those who developed diabetes had a significantly higher TyG (7.292 ± 0.611) than those who did not (6.756 ± 0.599; P < 0.001). Similarly, in the Japanese cohort, individuals with incident diabetes had higher TyG (7.026 ± 0.641) compared to those without (6.425 ± 0.640; P < 0.001).

### Age- and sex-specific incidence

**Figure 3:**
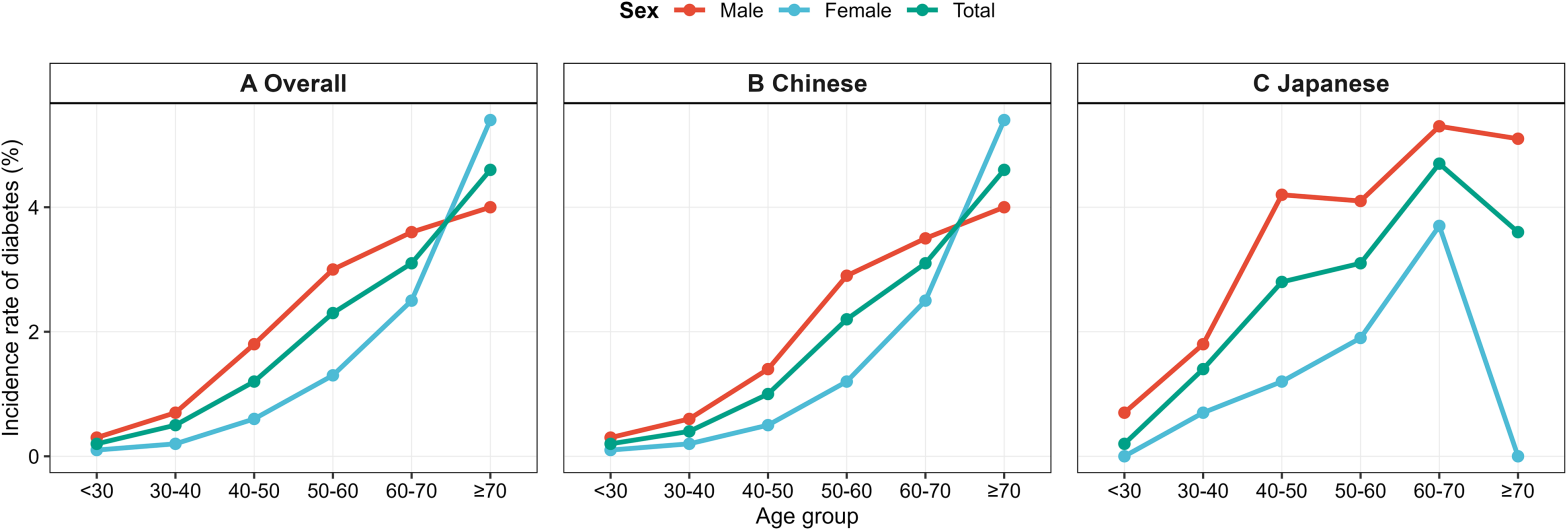
Age-specific diabetes incidence by sex and ethnicity. Incidence rates of diabetes across age categories (<30, 30–40, 40–50, 50–60, 60–70, ≥70 years) in **(A)** the total population, **(B)** the Chinese cohort, and **(C)** the Japanese cohort, stratified by sex.

Age- and sex-stratified incidence curves (**Figure 3**) revealed a consistent rise in diabetes risk with advancing age across all populations. Incidence increased modestly during early adulthood, accelerated after age 50, and peaked in individuals aged ≥70 years. Male individuals consistently exhibited higher diabetes incidence than female individuals in every age category. Japanese adults tended to display higher age-specific incidence, with the greatest divergence observed in middle-aged and older adults, and Japanese men aged ≥60 years demonstrated the highest incidence overall. The numbers of participants and incident cases in each age–sex stratum are shown in **Supplementary Table 4**. In Japanese women aged ≥70 years, no incident diabetes cases were observed (0/17), and this estimate should be interpreted cautiously due to the small subgroup size.

### Association between TyG and T2DM risk

Univariate Cox regression analysis (**Table 3**) identified multiple significant factors associated with incident diabetes. Age, BMI, SBP, DBP, FPG, TC, TG, LDL-c, ALT, AST, and smoking status were significant risk factors (all p<0.001), whereas HDL-c showed a significant protective effect. In addition, TyG exhibited a markedly elevated hazard ratio (HR = 3.731, 95%CI: 3.538–3.934, p<0.001), indicating a 3.7-fold increased risk per unit increment.

**Table 3:** The results of univariate analysis.

Variable	Statistics	HR	95% CI	P-value
Country				
Chinese	199050 (92.8%)	Ref.		
Japanese	15464 (7.2%)	0.269	(0.228, 0.317)	<0.001
Age (years)	41.9 ± 12.2	1.066	(1.063, 1.069)	<0.001
Gender				
Male	116462 (54.3%)	Ref.		
Female	98052 (45.7%)	0.513	(0.471, 0.558)	<0.001
Smoking status				
Non-smoker	165224 (77.0%)	Ref.		
Smoker	49290 (23.0%)	1.822	(1.683, 1.973)	<0.001
Drinking status				
Non-drinker	180348 (84.1%)	Ref.		
Drinker	34166 (16.0%)	1.062	(0.962, 1.173)	0.233
SBP (mmHg)	118.44 ± 16.1	1.039	(1.037, 1.041)	<0.001
DBP (mmHg)	73.8 ± 10.7	1.048	(1.045, 1.052)	<0.001
FPG (mmol/L)	4.9 ± 0.5	9.407	(8.609, 10.278)	<0.001
TC (mmol/L)	4.7 ± 0.9	1.348	(1.297, 1.401)	<0.001
TG(mmol/L)	1.3 ± 0.9	1.291	(1.276, 1.306)	<0.001
LDL-c (mmol/L) HDL-c(mmol/L)	2.7 ± 0.7 1.3 ± 0.3	1.225 0.354	(1.165, 1.287) (0.314, 0.400)	<0.001 <0.001
ALT (U/L)	23.4 ± 21.5	1.005	(1.004, 1.005)	<0.001
AST (U/L)	23.5± 12.0	1.007	(1.007, 1.008)	<0.001
TyG	8.3 ± 0.6	3.731	(3.538, 3.934)	<0.001

BMI, body mass index; FPG, fasting plasma glucose; DBP, diastolic blood pressure; TC, total cholesterol; SBP, systolic blood pressure; ALT, alanine aminotransferase; LDL-c, low-density lipid cholesterol; AST, aspartate aminotransferase; TyG, triglyceride–glucose index. HR, Hazard ratios; CI, confidence interval; Ref, reference.

### Results from a multivariate Cox proportional-hazards regression model

In multivariable models (**Table 4**), TyG remained independently associated with incident T2DM after sequential adjustment for demographic, lifestyle and metabolic factors. In the fully adjusted model (Model III), the HR per 1-unit increase in TyG was 2.315 (95% CI: 2.160–2.467) in the overall cohort, 2.258 (95% CI: 2.102–2.425) in Chinese and 2.074 (95% CI: 1.681–2.558) in Japanese (all P < 0.001).

**Table 4:** Relationship between TyG and the incident diabetes in different models.

TyG exposure	Overall (HR,95%CI, P)	Chinese (HR,95%CI, P)	Japanese (HR,95%CI, P)
Model I:			
TyG	3.731 (3.538, 3.934) <0.001	3.570 (3.373, 3.779) <0.001	3.760 (3.225, 4.383) <0.001
TyG Quartiles			
Q1	Ref.	Ref.	Ref.
Q2	2.414 (1.987, 2.934) <0.001	2.475 (1.966, 3.116) <0.001	1.991 (1.363, 2.909) =0.037
Q3	5.308 (4.443, 6.340) <0.001	5.491 (4.444, 6.785) <0.001	4.026 (2.856, 5.673) <0.001
Q4	13.524 (11.441,15.986) <0.001	13.924 (11.394, 17.016)<0.001	8.991 (6.538, 12.364) <0.001
P for trend	P<0.001	P<0.001	P<0.001
Model II:			
TyG	2.285 (2.141, 2.440) <0.001	2.192 (2.042, 2.353) <0.001	2.372 (1.966, 2.861) <0.001
TyG Quartiles			
Q1	Ref.	Ref.	Ref.
Q2	1.531 (1.258, 1.865) <0.001	1.547 (1.226, 1.951) =0.002	1.282 (0.870, 1.888) =0.201
Q3	2.324 (1.934, 2.793) <0.001	2.308 (1.858, 2.869) <0.001	1.981 (1.375, 2.855) =0.024
Q4	4.143 (3.465, 4.954) <0.001	4.069 (3.293, 5.027) <0.001	3.373 (2.351, 4.841) <0.001
P for trend	P<0.001	P<0.001	P<0.001
Model III:			
TyG	2.315 (2.160, 2.467) <0.001	2.258 (2.102, 2.425) <0.001	2.074 (1.681, 2.558) <0.001
TyG Quartiles			
Q1	Ref.	Ref.	Ref.
Q2	1.627 (1.335, 1.983) <0.001	1.641 (1.300, 2.071) =0.003	1.163 (0.785, 1.724) =0.451
Q3	2.563 (2.126, 3.090) <0.001	2.547 (2.044, 3.175) <0.001	1.688 (1.154, 2.469) =0.696
Q4	4.673 (3.885, 5.622) <0.001	4.675 (3.763, 5.809) <0.001	2.602 (1.761, 3.844) <0.001
P for trend	P<0.001	P<0.001	P<0.001

Model I: We did not adjust other covariates.

Model II: we adjusted age, gender, BMI, SBP, DBP, smoking and drinking status.

Model III: we adjusted age, gender, BMI, SBP, DBP, HLD-c, LDL-c, ALT, AST, smoking and drinking status.

HR, Hazard ratios; CI, confidence; Ref, reference; TyG, triglyceride–glucose index.

When TyG was modelled in quartiles, T2DM risk increased across categories (**Table 4**). Compared with Q1, adjusted HRs for Q4 were 4.673 (95% CI: 3.658–5.974) in the overall cohort, 4.675 (95% CI: 3.630–6.020) in Chinese and 2.602 (95% CI: 1.922–3.522) in Japanese (all P for trend < 0.001). Patterns were similar in less adjusted models, with some attenuation after multivariable adjustment.

### Kaplan–Meier survival analysis

**Figure 4:**
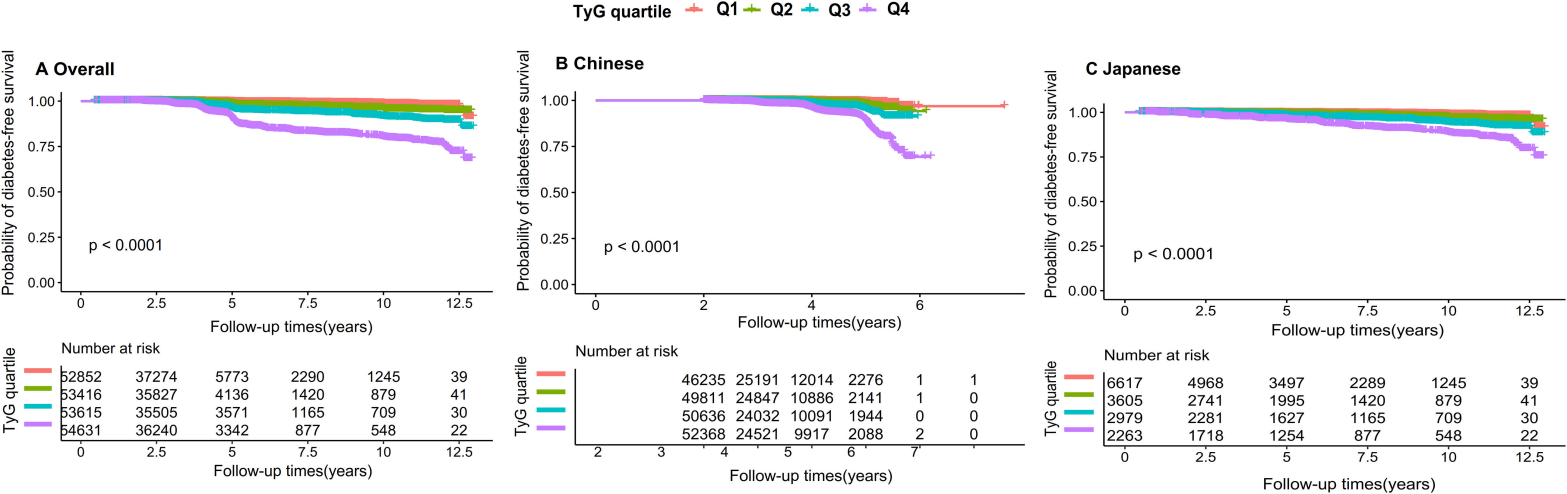
Kaplan–Meier diabetes-free survival across TyG. Kaplan–Meier curves showing diabetes-free survival according to TyG quartiles in **(A)** the total population, **(B)** the Chinese cohort, and **(C)** the Japanese cohort. Differences across quartiles were assessed using log-rank tests.

Kaplan–Meier curves for T2DM-free survival showed early and progressive separation across TyG quartiles (**Figure 4**). Participants in Q4 had the lowest probability of remaining T2DM-free during follow-up, whereas those in Q1 had the highest. Differences among curves were significant in the overall cohort and in each ethnic group (P <0.0001).

### Sensitivity analysis

Sensitivity analyses (**Table 5**) demonstrated that the association between TyG and incident diabetes was stable across multiple subgroup restrictions. After excluding participants living with obesity (BMI ≥ 28 kg/m²), higher TyG remained associated with incident T2DM with similar effect sizes. Comparable associations were observed when analyses were restricted to never-smokers, never-drinkers and participants with normal TG (<1.7 mmol/L). In all restricted subgroups, higher TyG quartiles were associated with higher T2DM risk, and P for trend across quartiles remained < 0.001. These results indicate that the TyG–T2DM association was robust across BMI categories, smoking and alcohol status, and baseline TG levels.

**Table 5:** Relationship between TyG and diabetes in different sensitivity analyses.

Exposure	Model I (HR,95%CI, P)	Model II (HR,95%CI, P)	Model III (HR,95%CI, P)	Model IV (HR,95%CI, P)
TyG	2.732 (2.535, 2.943) <0.001	2.330 (2.137, 2.540) <0.001	2.354 (2.184, 2.536) <0.001	3.950 (3.354, 4.698) <0.001
TyG (Quartile)				
Q1	Ref.	Ref.	Ref.	Ref.
Q2	1.783 (1.447, 2.196) <0.001	1.841 (1.453, 2.333) <0.001	1.669 (1.350, 2.065) <0.001	1.551 (1.271, 1.894) <0.001
Q3	3.074 (2.522, 3.747) <0.001	3.149 (2.515, 3.943) <0.001	2.665 (2.179, 3.260) <0.001	2.429 (2.005, 2.944) <0.001
Q4	6.092 (5.010, 7.409) <0.001	5.090 (4.058, 6.384) <0.001	4.719 (3.861, 5.767) <0.001	4.826 (3.895, 5.979) <0.001
P for trend	<0.001	<0.001	<0.001	<0.001

Model I shows a sensitivity analysis in participants with BMI <28 kg/m² (N = 197,661). We adjusted age, gender, SBP, DBP, HDL-c, LDL-c, ALT, AST, smoking and drinking status.

Model II shows a sensitivity analysis performed on never smoker participants (N = 165,224). We adjusted age, gender, BMI, SBP, DBP, HDL-c LDL-c, ALT, AST, and drinking status.

Model III shows a sensitivity analysis performed on never drinker participants (N = 180,348). We adjusted age, gender, BMI, SBP, DBP, HDL-c, LDL-c, ALT, AST, and smoking status.

Model IV shows sensitivity analysis in participants without TG≥1.7mmol/L (N = 169,329). We adjusted age, gender, BMI, SBP, DBP, HDL-c, LDL-c, ALT, AST, smoking and drinking status.

### Nonlinear associations and threshold

**Figure 5:**
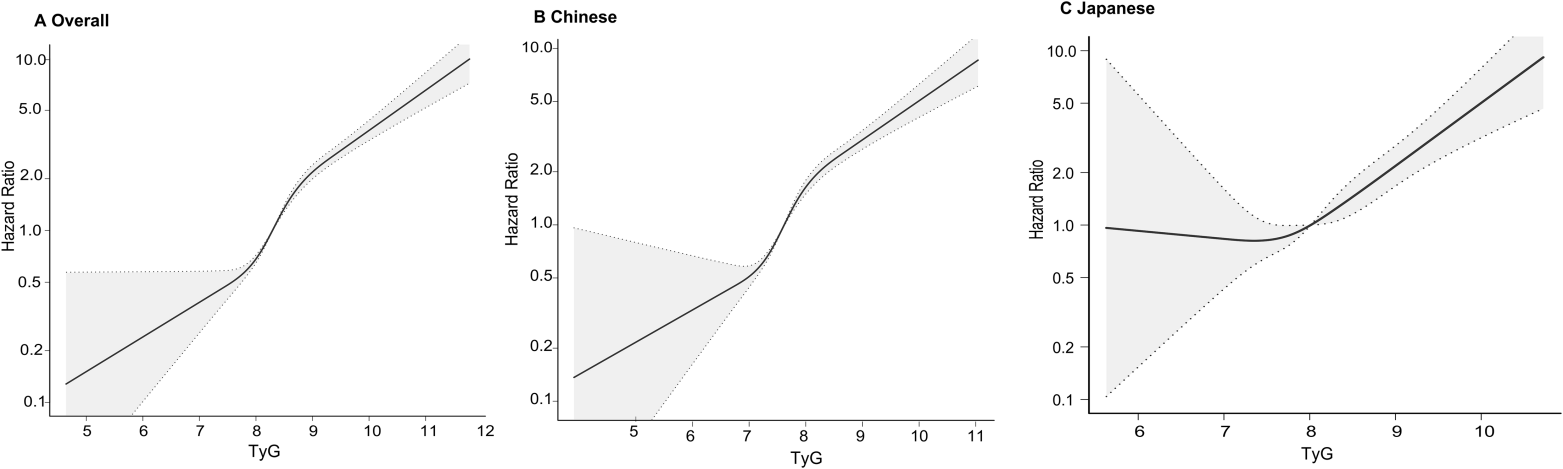
Nonlinear associations between TyG and diabetes risk. Restricted cubic spline models depicting the dose–response relationship between TyG and adjusted hazard ratios for incident T2DM in **(A)** the total population, **(B)** the Chinese cohort, and **(C)** the Japanese cohort. Shaded areas represent 95% confidence intervals.

Restricted cubic spline analyses suggested nonlinear associations between TyG and T2DM in the overall cohort and in both ethnic groups (**Figure 5**). T2DM risk increased with higher TyG, but the shape and slope of the curves differed between Chinese and Japanese. Two-piecewise Cox models identified population-specific inflection points (**Table 6**): TyG = 9.19 in the overall cohort, 8.98 in Chinese and 7.88 in Japanese. Below these values, the slope of the association was relatively shallow in Japanese and steeper in the overall and Chinese cohorts. Above the inflection points, T2DM risk increased more sharply in both ethnic groups, with particularly marked increases in Japanese. These findings indicate a nonlinear TyG–T2DM relationship with different threshold ranges in Chinese and Japanese.

**Table 6:** The result of the two-piecewise Cox regression model.

Incident diabetes	Overall (HR,95%CI, P)	Chinese (HR,95%CI, P)	Japanese (HR,95%CI, P)
Fitting model by standard Cox regression	2.315 (2.166, 2.475) <0.001	2.258 (2.102, 2.425) <0.001	2.074 (1.681, 2.558) <0.001
Fitting model by two-piecewise Cox regression			
Inflection point of TyG	9.19	8.98	7.88
TyG ≤ inflection point	2.927 (2.633, 3.254) <0.001	3.162 (2.749, 3.637) <0.001	1.024 (0.496, 2.116) =0.948
TyG > inflection point	1.526 (1.294, 1.801) <0.001	1.642 (1.431, 1.884) <0.001	2.287 (1.814, 2.884) <0.001
P for log-likelihood ratio test	<0.001	<0.001	0.064

We adjusted age, gender, BMI, SBP, DBP, HDL-C, LDL-c, ALT, AST, smoking and drinking status.

HR, Hazard ratios; CI, confidence; Ref, reference; TyG, The triglyceride-glucose (TyG) index.

### Subgroup analyses

Subgroup analyses (**Table 7**) showed broadly similar positive associations between TyG and incident T2DM across categories of age, sex, BMI, BP, smoking, alcohol and ethnicity. The relative risk associated with higher TyG tended to be larger in younger adults and in those with normal BMI, although no statistically significant interactions were detected (all P for interaction > 0.05). Overall, the direction and magnitude of the TyG–T2DM association were consistent across subgroups.

**Table 7:** Effect size of TyG on incident diabetes in prespecified and exploratory subgroups.

Characteristic	No of participants	HR (95%CI)	P value	P for interaction
**Age(years)**				0.0003
<45	139052	2.668	(2.383, 2.986)	<0.001
45-60	52529	2.377	(2.144, 2.637)	<0.001
≥60	22933	1.887	(1.664, 2.141)	<0.001
**Gender**				0.0144
Male	116462	2.215	(2.054,2.390)	<0.001
Female	98052	2.617	(2.326, 2.944)	<0.001
**BMI (kg/m^2^)**				<0.0001
<18.5	13190	2.155	(1.038, 4.475)	0.0395
18.5-24	121409	3.000	(2.674, 3.365)	<0.001
24-28	63062	2.239	(2.034, 2.464)	<0.001
≥28	16853	1.893	(1.666, 2.150)	<0.001
**Smoking status**				0.9515
Non-smoker	165224	2.319	(2.135, 2.519)	<0.001
Smoker	49290	2.310	(2.093, 2.550)	<0.001
**Drinking status**				0.7156
Non-drinker	180348	2.328	(2.164, 2.505)	<0.001
Drinker	34166	2.265	(1.979, 2.594)	<0.001
**SBP (mmHg)**				0.0005
<140	194765	2.487	(2.308, 2.681)	<0.001
≥140	19749	1.934	(1.704, 2.195)	<0.001
**DBP (mmHg)**				0.0496
<90	198447	2.385	(2.218, 2.564)	<0.001
≥90	16067	2.035	(1.759, 2.356)	<0.001
**Country**				0.5773
Chinese	199050	2.236	(2.082, 2.401)	<0.001
Japanese	15464	2.111	(1.740, 2.560)	<0.001

Note 1: Above model is adjusted for age, gender, BMI, SBP, DBP, HDL-c, LDL-c, ALT, AST, smoking and drinking status.

Note 2: In each case, the model is not adjusted for the stratification variable.

### Predictive performance

**Figure 6:**
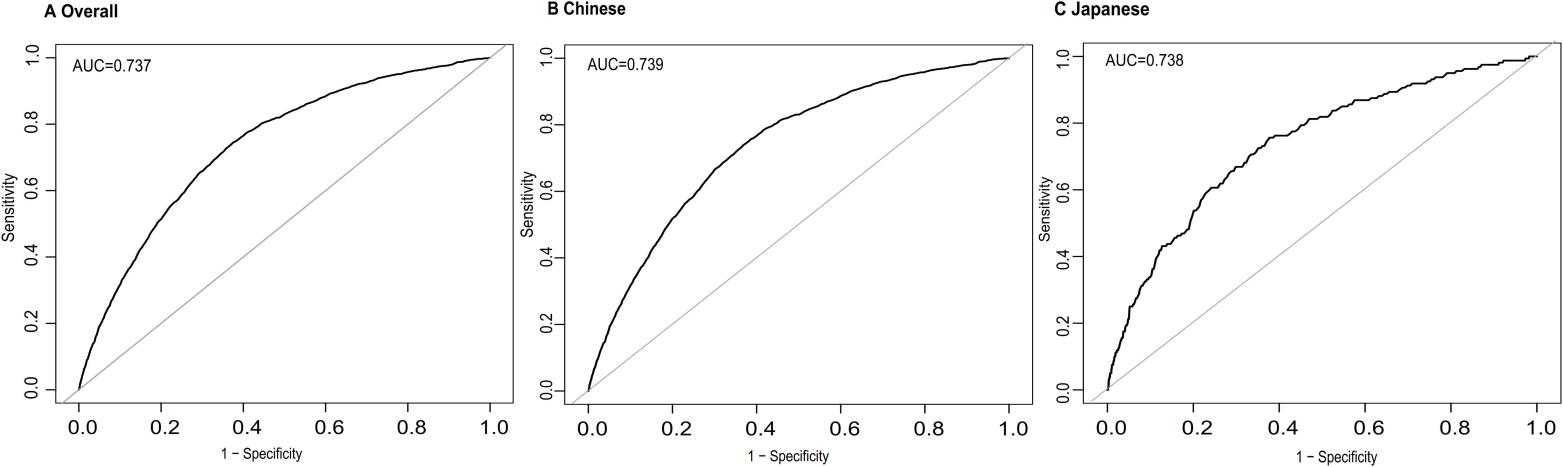
Predictive performance of the TyG for incident diabetes. Receiver operating characteristic **(ROC)** curves evaluating the predictive ability of the TyG for 5-year incident diabetes in **(A)** the total population, **(B)** the Chinese cohort, and **(C)** the Japanese cohort. Area under the curve **(AUC)** values are shown within each panel.

In receiver operating characteristic (ROC) analyses, TyG showed moderate and highly consistent discrimination for incident T2DM in the overall cohort and in both ethnic groups (**Figure 6**). The area under the curve (AUC) values were 0.737 in the overall cohort, 0.739 in Chinese participants, and 0.738 in Japanese participants, indicating similar predictive performance across East Asian populations despite differing baseline profiles and T2DM incidence. Using the Youden index, the optimal TyG cutoffs were 6.85 in the overall cohort (sensitivity 0.770, specificity 0.598) and in Chinese participants (sensitivity 0.782, specificity 0.587), and 6.65 in Japanese participants (sensitivity 0.725, specificity 0.642).

In supplementary ROC analyses directly comparing FPG, TG and TyG (**Supplementary Figure 1**), FPG consistently had the highest AUC, followed by TyG, with TG lowest. In the total population, AUCs were 0.773 for FPG, 0.732 for TyG and 0.697 for TG; similar patterns were seen in Chinese and Japanese. These results indicate that TyG improves prediction compared with TG alone and achieves a discrimination only modestly lower than FPG.

### Mediation analysis

Mediation analysis showed that BMI accounted for a modest proportion of the association between TyG and T2DM (**Table 8**). In the overall cohort, the proportion mediated by BMI was 19.70%, with corresponding estimates of 19.06% in Chinese participants and 14.22% in Japanese participants. Across all analyses, both the direct effect of TyG and the indirect effect through BMI were statistically significant, showing that the association between TyG and T2DM was only partly explained by BMI.

**Table 8:** Mediation analysis of the association between TyG and incident diabetes by BMI.

Effect	Total population	Chinese population	Japanese population
	HR (95% CI)	HR (95% CI)	HR (95% CI)
Direct effect (TyG→Diabetes)	2.30 (2.16-2.47)	2.25 (2.10-2.42)	2.07 (1.68-2.56)
Indirect effect (TyG→BMI→Diabetes)	1.22 (1.20-1.24)	1.20 (1.18-1.22)	1.12 (1.09-1.16)
Total effect (TyG→Diabetes)	2.75 (2.58-2.93)	2.66 (2.48-2.85)	2.34(1.90-2.88)
Proportion mediated	19.70%	19.06%	14.22%

The model was adjusted for multiple variables, including age, gender, SBP, DBP, HDL-c, LDL-c, ALT, AST, smoking, and drinking status.

TyG, The triglyceride-glucose (TyG) index; BMI, Body Mass Index; HR, Hazard Ratio; CI, Confidence Interval.

The proportion mediated refers to the percentage of the total effect of TyG on diabetes risk that is mediated through BMI. The analysis was performed using bootstrap methods to derive confidence intervals for the mediation proportion.

## Discussion

This large comparative cohort study of more than 214,000 adults demonstrated that higher TyG is strongly and independently associated with incident type 2 diabetes mellitus (T2DM) in both Chinese and Japanese populations. Across multivariable-adjusted models, individuals with elevated TyG values had substantially higher diabetes risk, and clear gradients in incidence were observed across TyG quartiles. The separation of baseline TyG distributions and Kaplan–Meier curves between participants who did and did not develop diabetes during follow-up suggests that TyG captures early metabolic changes that precede the onset of overt hyperglycaemia.

Beyond confirming its predictive value, our study also highlights ethnic heterogeneity in the TyG–diabetes association. Although higher TyG was associated with greater diabetes risk in both Chinese and Japanese adults, restricted cubic spline and two-piecewise Cox models revealed distinct patterns. In Chinese participants, diabetes risk increased almost continuously across the TyG spectrum, with only a modest change in slope around the identified threshold. By contrast, among Japanese adults, the TyG–diabetes curve was relatively flat at lower TyG values but rose steeply once TyG exceeded a lower threshold, suggesting a more threshold-like pattern of vulnerability. These divergences are consistent with established differences in body composition, fat distribution, and β-cell reserve across East Asian populations. Recent reviews and experimental studies indicate that Japanese adults often develop type 2 diabetes at relatively modest levels of adiposity, frequently with insufficient insulin secretion and a disproportionately high burden of visceral and ectopic fat (28, 29). Japanese cohort data further identify visceral fat accumulation as a key determinant of diabetes risk in this population (30). In our study, Japanese participants had lower BMI but more adverse lipid profiles and a higher prevalence of smoking and alcohol use, whereas Chinese participants exhibited higher BMI and blood pressure (**Supplementary Table 2**), pointing to distinct constellations of metabolic stressors. These clinical patterns align with national nutrition surveys showing that Japanese diets remain relatively richer in fish and marine-derived n−3 fatty acids (31), whereas Chinese diets are more carbohydrate-dense with greater reliance on refined grains and staple white rice (32). Taken together, these observations support a model in which diabetes risk in Japanese adults rises sharply once lipid- and visceral fat–related stress exceeds a critical threshold—consistent with the threshold-like TyG–diabetes curve—whereas in Chinese adults a broader accumulation of adiposity and cardiometabolic burden may underlie the smoother, more cumulative TyG–diabetes gradient. Because our cohorts did not include direct measures of visceral or liver fat, genetic variants, or detailed dietary intake, these mechanistic interpretations should be regarded as hypothesis-generating rather than definitive.

The mediation analysis provides further insight into the pathways linking TyG with diabetes. BMI was specified a priori as the mediator of overall adiposity because it was the only adiposity measure collected in a harmonised manner across both cohorts, whereas waist circumference, waist–hip ratio, and imaging-based assessments of visceral or hepatic fat were either unavailable or inconsistently measured. BMI accounted for only a modest proportion (approximately 15–20%) of the overall TyG–diabetes association in the combined cohort and within each ethnic group. Although the mediation estimates differed numerically between Chinese and Japanese adults, the study was not specifically designed or powered to formally compare these indirect effects between ethnic groups, and no formal statistical test was performed; therefore, these differences should be interpreted descriptively rather than as evidence of true between-population differences. Because TyG is calculated from TG and FPG, it reflects both glycaemic burden and lipid-driven insulin resistance rather than adiposity alone. The modest proportion of the association mediated through BMI therefore suggests that TyG also captures pathways related to ectopic fat accumulation, hepatic steatosis, and early insulin resistance that are not fully represented by BMI (8, 33). Taken together, the consistently limited mediation by BMI in both cohorts supports our conclusion that TyG reflects metabolic pathways that are not adequately captured by simple anthropometric measures.

Consistent with this interpretation, BMI-stratified analyses showed that the relative hazards associated with higher TyG were evident across all BMI categories but were strongest among participants in the normal-BMI range (18.5–24 kg/m²), with somewhat attenuated yet still positive gradients at higher BMI levels. This pattern suggests that BMI may act not only as a partial mediator but also as an effect modifier of the TyG–diabetes relationship. Clinically, it underscores the particular value of TyG for identifying metabolically unhealthy individuals who would not be flagged by adiposity-based screening alone—namely, those with “normal weight” but substantial visceral or ectopic fat accumulation—who may benefit from earlier monitoring and intervention once elevated TyG is detected.

The inclusion of FPG in the TyG formula naturally raises the concern that its predictive value might simply mirror that of FPG alone. As shown in **Supplementary Figure 1**, FPG had the highest AUC for incident diabetes, whereas TyG showed very similar discrimination and performed better than TG alone. This pattern suggests that TyG functions primarily as a glycaemic marker while also integrating a modest additional contribution from TG. Elevated TG levels are a hallmark of insulin-resistant states and hepatic fat accumulation, which typically develop before overt hyperglycaemia (34), and their contribution is reflected in the TyG construct. Consistent with this, TyG remained strongly associated with incident diabetes among participants with normal BMI and in multiple low-risk subgroups, suggesting that it captures lipid- and insulin resistance–related risk that is not fully represented by glycaemia or adiposity alone. Previous cohort studies have likewise reported that TyG improves diabetes risk stratification among individuals with normal fasting glucose (35).

Placing our findings in a broader international context highlights clear interethnic differences in TyG-based risk thresholds. As summarized in **Supplementary Table 5**, several non-Asian and multi-ethnic cohorts have reported relatively high optimal TyG cut-off values for the detection or prediction of diabetes or insulin resistance, generally higher than those observed in our Chinese and, in particular, Japanese adults (35–38). These interethnic differences likely reflect broader metabolic patterns. At a given BMI, East Asian populations typically have a higher body-fat percentage and a more unfavorable, visceral-dominant fat distribution than people of European descent and therefore develop insulin resistance and type 2 diabetes at substantially lower levels of adiposity (39, 40).

Taken together, our findings indicate that the TyG is a practical and informative marker for diabetes risk screening in East Asian populations. TyG showed stable, graded associations with incident diabetes in both cohorts, including among participants with normal BMI and in multiple low-risk subgroups, and mediation analyses suggested that only a modest proportion of this relationship is explained by BMI. Thus, TyG appears to capture lipid- and insulin resistance–related risk that is not fully represented by glycaemia or adiposity alone and can complement, rather than replace, traditional anthropometric measures. In addition, the ethnic differences in non-linear patterns and thresholds observed between Chinese and Japanese adults indicate that TyG-based risk-stratification strategies should be calibrated to specific populations rather than based on a single universal cut-off.

Strengths of this study include the large sample size across two East Asian populations, the harmonized analytic framework applied to both cohorts, and the generally consistent results after extensive covariate adjustment and multiple sensitivity analyses. Nonetheless, several limitations should be acknowledged. First, key anthropometric and visceral adiposity indicators—such as waist circumference, waist–hip ratio, and imaging-based fat measures—were not collected in a standardized, comparable way across cohorts, so our cross-population evaluation focused on biochemical markers that were measured uniformly in both datasets, limiting direct comparison between TyG and anthropometric indices. Second, residual confounding from unmeasured lifestyle, genetic, or environmental factors cannot be excluded, and the smaller Japanese cohort may have reduced the precision of ethnic-specific estimates. Finally, the observational design precludes causal inference, and the associations reported here should be interpreted as indicative of risk rather than proof of causality.

In conclusion, while the triglyceride-glucose (TyG) index is a valuable tool for identifying individuals at risk for diabetes, our study highlights that the association between TyG and diabetes risk is significantly mediated by body mass index (BMI). This underscores the critical role of adiposity in this relationship and suggests that BMI should be considered as a key mediating factor when evaluating diabetes risk. Rather than replacing traditional markers such as BMI, TyG should be used alongside them to provide a more comprehensive risk assessment. Incorporating BMI into risk-stratification frameworks—along with TyG and other glycaemic and anthropometric measures—can improve the early detection of high-risk individuals, particularly those with normal BMI but unhealthy metabolic profiles, and facilitate more targeted and effective prevention strategies.

## Data Availability

The original contributions presented in the study are included in the article/**Supplementary Material**. Further inquiries can be directed to the corresponding author.
