# Supplementary material for: Association between the triglyceride-glucose index and risk of type 2 diabetes mellitus and the mediating effect of BMI: a comparative analysis in Chinese and Japanese populations
Source: Front Endocrinol (Lausanne). 2026 Mar 2;17:1701371. doi: 10.3389/fendo.2026.1701371 (PMC12989386; doi:10.3389/fendo.2026.1701371)

**Association Between The triglyceride-glucose (TyG) index and Risk of Type 2 Diabetes Mellitus and the Mediating Effect of BMI: A Comparative Analysis in Chinese and Japanese Populations**

Running title: Insights from Population-Specific Thresholds and Differential Mediation Pathways in East Asian Cohorts

**Yuxian Chen^1,2#^, Haiyong Zeng^1#^, ZiQi Luo^4#^, Haofei Hu^*3^, XinYu Wang^*1,2^**

^1^Department of Endocrinology and Metabolism, The First Affiliated Hospital of Shenzhen University, Shenzhen 518000, Guangdong Province, China

^2^School of Medicine Shenzhen University, Shenzhen 518000, Guangdong Province, China

^3^Department of Nephrology, Shenzhen Second People’s Hospital, Shenzhen 518000, Guangdong Province, China

^4^Department of General Practice, Shenzhen Second People’s Hospital, Shenzhen 518000, Guangdong Province, China

***Corresponding author**

**Haofei Hu**

Department of Nephrology,

Shenzhen Second People’s Hospital,

No.3002 Sungang Road, Futian District,

Shenzhen 518000,

Guangdong Province,

China

Tel:+86-755-83366388

E-mail: [huhaofei0319@126.com](mailto:huhaofei0319@126.com)

***Corresponding author**

**XinYu Wang**

Department of Endocrinology and Metabolism

Shenzhen Second People’s Hospital,

No.3002 Sungang Road, Futian District,

Shenzhen 518000,

Guangdong Province,

China

<Tel:+86-755-83366388>

E-mail:[wxyhorse@126.com](mailto:wxyhorse@126.com)

**Supplementary Table 1. Collinearity diagnostics steps.**

| Variable | VIF  Step 1 | Step 2 |
| --- | --- | --- |
|  |  |  |
| Gender | 1.4 | 1.4 |
| Age(years) | 1.3 | 1.3 |
| Smoking status | 1.2 | 1.2 |
| Drinking status | 1.1 | 1.1 |
| ALT(U/L) | 3.4 | 3.4 |
| AST(U/L) | 3.1 | 3.1 |
| TC(mmol/L) | 8.6 | NA |
| LDL-c(mmol/L)  HDL-c(mmol/L) | 6.3  1.8 | 1.2  1.2 |
| BMI(kg/m^2^) | 1.6 | 1.6 |
| SBP(mmHg) | 2.2 | 2.2 |
| DBP(mmHg) | 2.1 | 2.1 |
| TyG | 2.1 | 1.6 |

BMI, Body mass index; SBP, Systolic blood pressure; DBP, Diastolic blood pressure; ALT, Alanine aminotransferase; AST, Aspartate aminotransferase; TC, Total cholesterol; LDL-c, Low-density lipid cholesterol; FPG, Fasting plasma glucose; TyG ,The triglyceride-glucose index

Abbreviation: VIF: variance inflation factor; VIF = 1/(1-R^2^).

Note: The variables with VIF>5 will be regarded as collinear variables and cannot be included in the multiple regression model.

**Supplementary Table 2. Demographic and clinical characteristics of study population by the country**

| Characteristics | Chinese (n=199,050) | Japanese(n=15,464) | *P*-value |
| --- | --- | --- | --- |
| Demographics |  |  |  |
| Age (years) | 41.8 ± 12.5 | 43.7 ± 8.9 | <0.001 |
| Gender, n (%) |  |  | <0.001 |
| Male | 108032 (54.3%) | 8430(54.5%) |  |
| Female | 91018 (45.7%) | 7034(45.5%) |  |
| Lifestyle Factors |  |  |  |
| Smoking, n (%) |  |  | <0.001 |
| No | 156193 (78.5%) | 9031 (58.4%) |  |
| Yes | 42857 (21.5%) | 6433 (41.6%) |  |
| Drinking, n (%) |  |  | <0.001 |
| No | 168543 (84.7%) | 11805 (76.3%) |  |
| Yes | 30507 (15.3%) | 3659 (23.7%) |  |
| Anthropometric Measures |  |  |  |
| BMI (kg/m²) | 23.2 ± 3.3 | 22.1 ± 3.1 | <0.001 |
| SBP (mmHg) | 118.7 ± 16.2 | 114.5 ± 15.0 | <0.001 |
| DBP (mmHg) | 74.0 ± 10.7 | 71.6 ± 10.5 | <0.001 |
| Laboratory Parameters |  |  |  |
| FPG (mmol/L) | 4.9 ± 0.5 | 5.2 ± 0.4 | <0.001 |
| TC (mmol/L) | 4.7 ± 0.9 | 5.1 ± 0.9 | <0.001 |
| TG (mmol/L) | 1.10(1.10-1.10) | 0.8(0.7-0.8) | <0.001 |
| HDL-C (mmol/L) | 1.4 ± 0.3 | 1.5 ± 0.4 | <0.001 |
| LDL-C (mmol/L) | 2.7 ± 0.7 | 3.2 ± 0.8 | <0.001 |
| ALT (U/L) | 19.4 (19.4-19.5) | 17.5 (17.3-17.6) | <0.001 |
| AST (U/L) | 22.0(21.9-22.03) | 17.3 (17.2-17.4) | <0.001 |
| TyG | 8.4 ± 0.6 | 8.0 ± 0.6 | <0.001 |

Values are n (%), mean±SD or medians (quartiles)

BMI, body mass index; FPG, fasting plasma glucose; DBP, diastolic blood pressure; TC, total cholesterol; SBP, systolic blood pressure; TG, triglyceride; ALT, alanine aminotransferase; LDL-c, low-density lipid cholesterol; AST, aspartate aminotransferase; HDL-c, high-density lipoprotein cholesterol; TyG, The triglyceride-glucose index

**Supplementary Table 3. Baseline TyG by incident diabetes during follow-up, stratified by cohort**

| **Country** | **Incident diabetes during follow-up** | **Participants** | **Baseline TyG** | **P value** |
| --- | --- | --- | --- | --- |
| **Chinese** | No incident diabetes  Incident diabetes | 196,860  2190 | 6.756 ± 0.599  7.292 ± 0.611 | <0.001  <0.001 |
| **Japanese** | No incident diabetes  Incident diabetes | 15,091  373 | 6.425 ± 0.640  7.026 ± 0.641 | <0.001  <0.001 |

**Supplementary Table 4. Number of incident diabetes cases (participants) by age group and sex in the Chinese and Japanese cohorts**

| **Age group** | **Sex** | **Chinese(N)** | **Japanese(N)** | **overall (N)** |
| --- | --- | --- | --- | --- |
| **<30** | male | 17(12,204) | 1(143) | 18(12,347) |
|  | female | 40(15,027) | 0(273) | 40(15,300) |
| **30-40** | male | 78(36,366) | 53(2,874) | 131(39,340) |
|  | female | 262(42,976) | 17(2,301) | 279(45,277) |
| **40-50** | male | 104(20,671)) | 129(3,054) | 233(23,724)) |
|  | female | 318(22,057) | 34(2,733) | 352(24,790)) |
| **50-60** | male | 146(11,864) | 78(1,884) | 224(13,748) |
|  | female | 449(15,564)) | 28(1,491) | 477(17,055) |
| **60-70** | male | 182(7,233) | 23(437) | 205(7,670) |
|  | female | 301(8,562) | 8(219) | 309(8,781) |
| **≥70** | male | 140(2,580) | 2(39) | 142(2,619) |
|  | female | 153(3,846) | 0(17) | 153(3,863)) |

**Supplementary Table 5. Reported TyG thresholds for diabetes or insulin resistance in non-Asian and multi-ethnic cohorts.**

| **First author (Year)** | **Country/ethnicity** | **TyG cut-off** | **AUC (95% CI)** |
| --- | --- | --- | --- |
| **Campos Muñiz, C(2023)** | Mexico | Female:9.45  Male:9.12 | 0.934(0.924-0.924)  0.824(0.824-0.873) |
| **Navarro González, D**  **(2016)** | White European | ≥8.31 | 0.75(0.70–0.81) |
| **D'Elia, L(2025)** | Italian men | 4.88 | 0.75 |
| **Rhaiem, TB(2025)** | Caucasia | 8.31 | 0.77 |

**Supplementary Figure 1.**


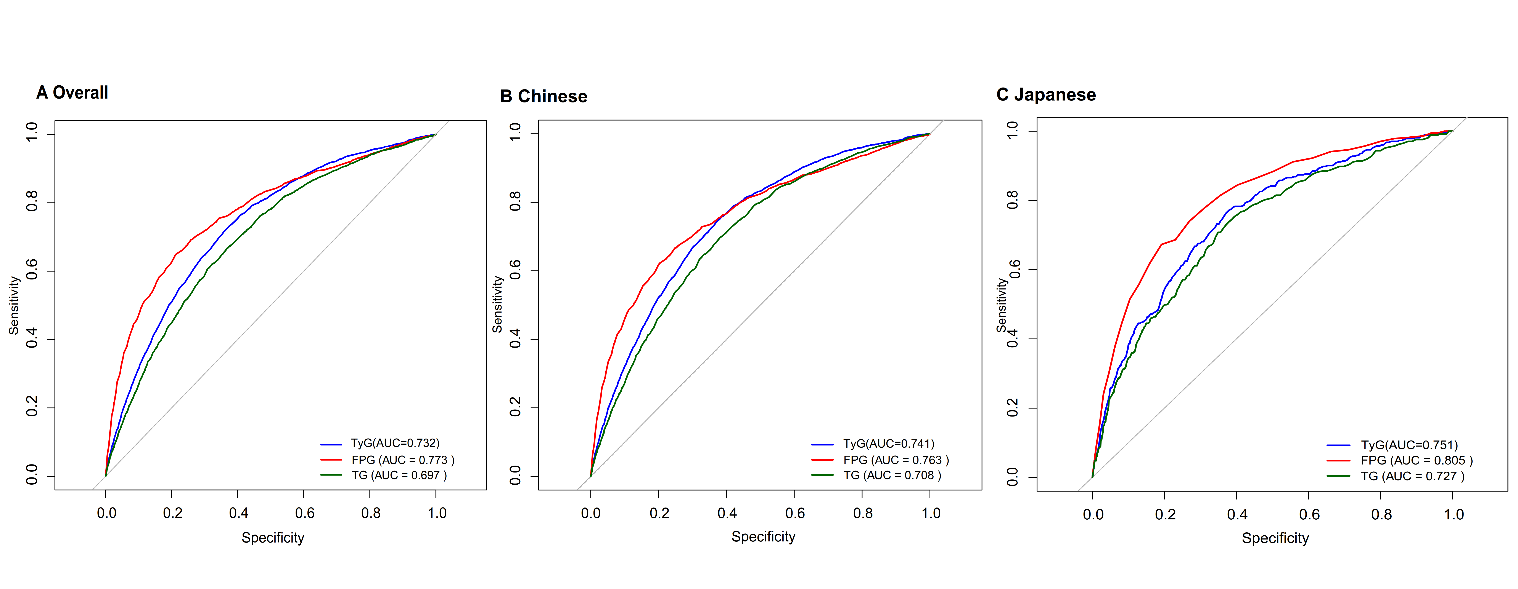

Supplement: Supplementary file 1 [file Table1.docx]
